# Cytokinin Production by *Azospirillum brasilense* Contributes to Increase in Growth, Yield, Antioxidant, and Physiological Systems of Wheat (*Triticum aestivum* L.)

**DOI:** 10.3389/fmicb.2022.886041

**Published:** 2022-05-19

**Authors:** Muhammad Saqlain Zaheer, Hafiz Haider Ali, Muhammad Arslan Iqbal, Kehinde O. Erinle, Talha Javed, Javaid Iqbal, Makhdoom Ibad Ullah Hashmi, Muhammad Zahid Mumtaz, Ehab A. A. Salama, Hazem M. Kalaji, Jacek Wróbel, Eldessoky S. Dessoky

**Affiliations:** ^1^Department of Agricultural Engineering, Khwaja Fareed University of Engineering and Information Technology, Rahim Yar Khan, Pakistan; ^2^Sustainable Development Study Center (SDSC), Government College University, Lahore, Pakistan; ^3^District Headquarter Hospital, Muzaffargarh, Pakistan; ^4^School of Agriculture, Food and Wine, The University of Adelaide, Adelaide, SA, Australia; ^5^College of Agriculture, Fujian Agriculture and Forestry University, Fuzhou, China; ^6^Department of Agronomy, University of Agriculture, Faisalabad, Pakistan; ^7^Department of Entomology, Muhammad Nawaz Shareef (MNU) University of Agriculture, Multan, Pakistan; ^8^Department of Chemical Engineering, Khwaja Fareed University of Engineering and Information Technology, Rahim Yar Khan, Pakistan; ^9^Institute of Molecular Biology and Biotechnology (IMBB), The University of Lahore, Lahore, Pakistan; ^10^Agricultural Botany Department, Faculty of Agriculture Saba Basha, Alexandria University, Alexandria, Egypt; ^11^Department of Plant Physiology, Institute of Biology, Warsaw University of Life Sciences SGGW, Warsaw, Poland; ^12^Institute of Technology and Life Sciences - National Research Institute, Raszyn, Poland; ^13^Department of Bioengineering, West Pomeranian University of Technology in Szczecin, Szczecin, Poland; ^14^Department of Biology, College of Science, Taif University, Taif, Saudi Arabia

**Keywords:** bacteria, cytokinin, hormones, rhizosphere, wheat

## Abstract

Plant growth-promoting rhizobacteria are known to associate with several cereal crops. The rhizobacterium exerts its function by synthesizing diverse arrays of phytohormones, such as cytokinin (Ck). However, it is difficult to determine the plant growth promotion when a bacterium produces many different kinds of phytohormones. Therefore, to assess the involvement of Ck in growth promotion and activation of antioxidant and physiological systems, we set up this experiment. Wheat seeds (*Triticum aestivum* L.) were inoculated with *Azospirillum brasilense* RA−17 (which produces zeatin type Ck) and RA−18 (which failed to produce Ck). Results showed that seed inoculation with RA−17 significantly improved growth and yield-related parameters compared with RA−18. The activity of enzymes, proline contents, and endogenous hormonal levels in wheat kernels were improved considerably with RA−17 than with RA−18. Strain RA−17 enhanced grain assimilation more than strain RA−18 resulting in a higher crop yield. These results suggest that microbial Ck production may be necessary for stimulating plant growth promotion and activating antioxidant and physiological systems in wheat.

## Introduction

Rhizobacteria play a vital role in ecosystem services. The bacterial population around plant roots is more versatile in nutrients solubilization, transformation, and mobilization (Hussain et al., 2013; Sabir et al., 2013), thus increasing plant growth (Qureshi et al., 2012; Liu et al., 2019). It is carried out by synthesizing diverse arrays of plant hormones. such as auxin, cytokinin (Ck), gibberellin, abscisic acid (ABA), and indole-3-acetic acid (IAA) (Ahmed and Hasnain, 2020; Ortiz-Castro et al., 2020). Microbe-derived phytohormones influence plant growth parameters and have been well studied (Arshad and Frankenberger, 1991; Zafar et al., 2012; Farooq and Bano, 2013; Xu et al., 2018). Phytohormones improve the metabolic functions of plants, influence root exudation, alter gene expressions, cell division, xylem processes, root development, and different signaling processes (Campos et al., 2019; Mushtaq et al., 2019).

Cytokinins play a significant role in plant growth promotion in different mechanistic ways, such as cell division, chlorophyll accumulation, expansion of leaves, by delaying leaf senescence, and etioplasts conversion to the chloroplast (Angeli et al., 2001; Zafar et al., 2012; Zwack and Rashotte, 2013). Both plants and microorganisms produce Cks, which differ in types and activity (Akhtar et al., 2020). Cerný et al. (2010) noticed that the different kinds of proteins and phosphor-proteins are present in the chloroplast, a direct singling chain responsible for the CK actions in the chloroplast. Rhizobacterial inoculation with the seeds was independent of specific hormone signaling. For example, López-Bucio et al. (2007) reported that *Bacillus megaterium* inoculation enhanced biomass and stimulated the growth of roots in auxin mutant Arabidopsis by auxin and ethylene-independent signaling system. Ortíz-Castro et al. (2008) found reduced growth-promoting effects in Arabidopsis inoculation with *B*. *megaterium*. They suggested that crop growth stimulation by the microbe may require an intact Ck signaling pathway. Rice seedling inoculation with a Ck-mutant *Magnaporthe oryzae* did not show distinct growth and development features until an exogenous source of Ck was applied (Chanclud et al., 2016).

Wheat (*Triticum aestivum* L.) is a significant food source for human consumption. For decades, improving growth and increasing yield have been the major interest among wheat breeders (Sears, 1997; Zaheer et al., 2021b). It has been suggested that wheat production needs to be increased by an estimated 60% to meet market demand in the next 10 years (Zaheer et al., 2019a). Interactions between (Ck producing) rhizobacteria and wheat is crucial for promoting growth and increasing yield. Zaheer et al. (2019a) reported that Ck plays a vital role in improving wheat yield. Its production with the rhizobacteria strain can be more beneficial for the physiological processes of the wheat plant. The use of specific bacterial strains that can produce more Ck in the plant can benefit the wheat plant's internal metabolic and growth improvement processes. Little study is available for those rhizobacteria that can make Ck, so there is a need to investigate the Ck-producing bacteria and their effect on plant growth (Sarkar et al., 2018).

In this study, carried out in the field, wheat seeds were inoculated with a strain of Ck (zeatin)-producing bacteria, *Azospirillum brasilense*, and its effects on wheat growth, physiological and antioxidant systems were compared to inoculation with a Ck non-producing strain of the same bacteria. We hypothesize that Ck-producing bacteria, compared with non-producing bacteria, are more beneficial for wheat growth and yield improvement and upregulation of physiological and antioxidant systems.

## Materials and Methods

### Bacterial Strains and Preparation of Wheat Seeds

**Figure 1:**
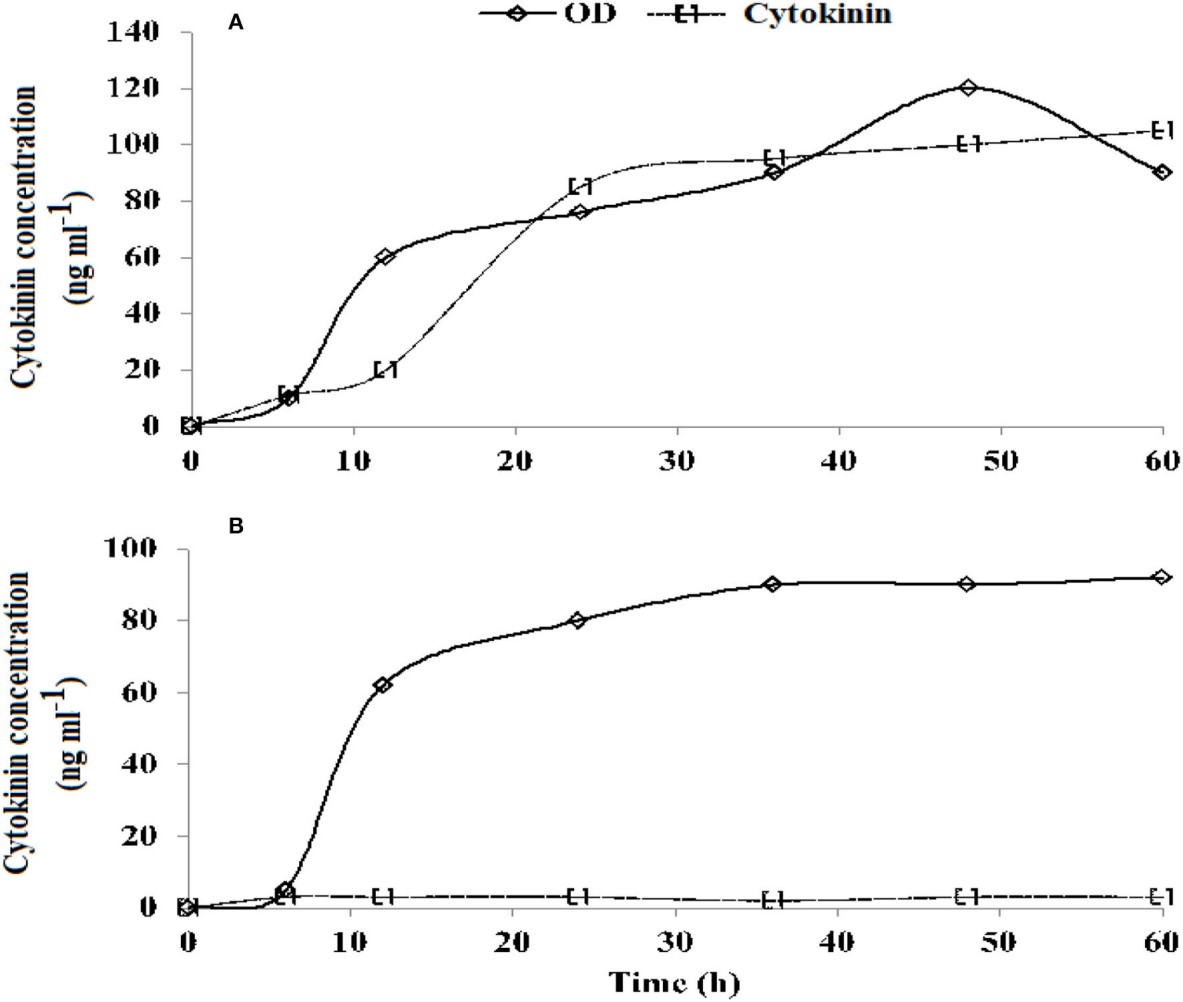
Growth curve (OD, optical density) and cytokinin (Ck accumulation) of a cultured medium of *Azospirillum brasilense* RA−17 **(A)** and RA−18 **(B)**.

Cytokinin producing (RA−17) and non-producing (RA−18) strains of *A. brasilense* were obtained from the soil lab of the Ayub Agricultural Research Institute, Faisalabad. Ck production was confirmed before being used for the experiments (Figure 1). Surface sterilized seed with ethanol (70%) and sodium hypochlorite of an approved wheat variety “Galaxy−2013” were used in the experiment. Seeds were inoculated with either the Ck producing or non-producing strain (10 × 10^8^ CFU ml^−1^ obtained at exponential growth phase) following the procedure reported by Fukami et al. (2016).

### Plant Growth Conditions

Field experiments were conducted in the semi-arid region of Bahawalpur from 2017 to 2018. The experimental plots size was 5 × 5 m. The soil was loamy, having 0.61% organic matter, 8.1 pH, 249 μS cm^−1^ electrical conductivity, 7.08 ppm available P, 112 ppm available K, and 34% saturation percentage. The Galaxy−2013 wheat variety, released by Punjab Seed Corporation in 2013 and mostly grown in the area of Bahawalpur, was used for the experiment.

To compare the effects of Ck-producing strains with non-producing strains on wheat growth and physiological parameters, three treatments, uninoculated control (T_0_), inoculation with Ck producing (T_1_), and non-Ck producing (T_2_) *A. brasilense*, were arranged in Randomized Complete Block Design (RCBD). Wheat seeds (according to the treatments) were sown on 15 November 2017. According to the recommendations, recommended fertilizers (120-60-60 NPK kg/ha) and four irrigation schemes (3 Acr inches) were applied. Briefly, 3 feet was the inter-plot distance in all replications.

### Measured Parameters

Physiological parameters were determined using 20 plants from each plot with three replications and an average. Different growth and yield-related parameters were measured by standard procedures (Bremner, 1965; Tkachuk, 1966; Ullah et al., 2018). Crop growth rate (CGR) and relative growth rate (RGR) were calculated by the formula described by Karimi and Siddique (1991), and net assimilation rate (NAR) was obtained by following the procedure of Vernon and Allison (1963). The leaf area index (LAI) was noticed using the digital leaf area meter, and the chlorophyll meter was used to detect the chlorophyll content. An infrared gas analyzer (IRGA) was used to notice the stomatal conductance and photosynthetic rate. Epicuticular wax was determined by following the approach described by Silva-Fernandes et al. (1964). Relative water content (RWC) of a leaf was measured according to Barrs and Weatherley (1962) equation:


$$\begin{array}{l} \text{RWC }(\%)\text{=}(\text{Fresh weight}-\text{dry weight})\text{ / }(\text{turgid weight}\\ \;-\text{dry weight})\;\times \;\text{100} \end{array}$$

According to Kudoyarova et al. (2014), levels of endogenous hormones in kernels of wheat were noticed. Proline content determined in wheat leaves by the ninhydrin method as reported by Bates et al. (1973). The activity of antioxidant enzymes, such as Ascorbate peroxidase (APX), catalase (CAT), Peroxidase (POD), and superoxide dismutase (SOD) was determined according to Nakano and Asada (1981), Vanacker et al. (2000), Ghanati et al. (2002), and Beyer and Fridovich (1987), respectively.

### Statistical Analysis

Data from three experiments were analyzed using Fisher's test, and least significant difference (LSD) was used to compare treatments at a 95% probability level (Steel et al., 1997).

## Results

*Azospirillum brasilense* strain RA−17 produced an increasingly higher concentration of Ck, while the RA−18 strain consistently delivered a very low concentration of Ck (Figure 1).

### Growth and Yield-Related Parameters

**Figure 2:**
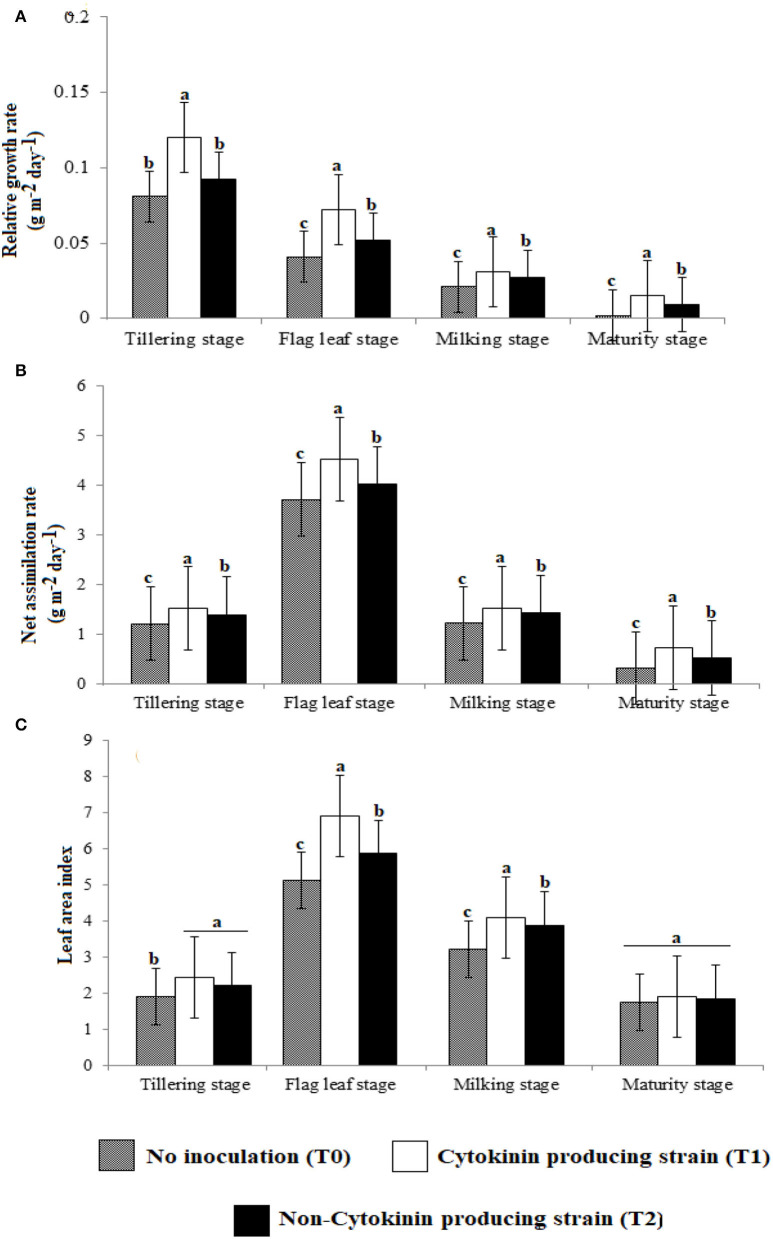
Effect of Ck-producing *A. brasilense* RA−17 and non-producing *A. brasilense* RA−18 on **(A)** Relative growth rate (RGR), **(B)** Net assimilation rate (NAR), and **(C)** Leaf area index (LAI) of wheat plants. Different letters are significantly other in each column (*p* ≤ 0.05), and the different letter shows a significant difference in treatments at a 5% probability level.

Relative growth rate (Figure 2) and net assimilation rate (Figure 2) were higher in wheat plants inoculated with Ck-producing than non-producing strain at tillering, flag leaf, milking, and maturity stages, and were lowest in the uninoculated plants. RGR was highest at the tillering stage and was about two-folds higher than at the flag leaf stage, about four-folds higher than at the milking stage, and six-folds higher than at the maturity stage. NAR was highest at the flag leaf stage and was about two-folds higher than at tillering and milking stages, which did not differ, but was four–six-folds more increased than at the maturity stage.

At tillering stage, the LAI did not differ between plants inoculated with Ck-producing and non-producing bacterial strains but it was lowest in the uninoculated plants (Figure 2). However, at the flag leaf and milking stage, LAI was more significant with the Ck-producing than non-producing strain and was lowest in the uninoculated plants. LAI was highest at the flag leaf stage and was about two-folds higher than at the milking stage. A non-significant difference was noticed in LAI at the maturity of all treatments.

Crop growth rate at tillering and flag leaf stages was greater in wheat plants inoculated with the Ck-producing than non-producing strain, and it was lowest in the uninoculated plants (Table 1). CGR was about four-folds higher in wheat at the flag leaf stage than at the tillering stage. All growth and yield-related parameters were more significant with the Ck-producing than non-producing bacterial strain and were lowest in the uninoculated plants (Table 2).

**Table 1:** Effect of cytokinin producing *Azospirillum brasilense* RA−17 (T_1_) and non-producing *A. brasilense* RA−18 (T_2_) on crop growth rate (CGR, g m^−2^ day^−1^) of wheat.

**Treatment**	**Tillering stage**	**Flag leaf stage**
T_0_ (control)	1.73 c	6.33 c
T_1_	2.15 a	9.58 a
T_2_	1.94 b	8.44 b

*The different letter shows a significant difference in treatments at a 5% probability level*.

**Table 2:** Effect of cytokinin (Ck)-producing *A. brasilense* RA−17 (T_1_) and non-producing *A. brasilense* RA−18 (T_2_) on plant height (cm) and yield parameters of wheat.

**Treatments**	**Plant height (cm)**	**Spike length (cm)**	**Number of spikelets per spike**	**Number of grains per spike**	**1,000-grain weight (g)**	**Grain yield (kg/ha)**
T_0_ (Control)	83.33 c	8.88 c	14.23 c	24.34 c	37.7 c	3,850 c
T_1_	88.34 a	11.19 a	19.23 a	28.54 a	44.12 a	4,666 a
T_2_	85.53 b	10.06 b	18.55 b	26.52 b	41.56 b	4,182 b

*The different letter shows a significant difference in treatments at a 5% probability level*.

### Physiological and Photosynthetic Parameters in Wheat Leaves

**Figure 3:**
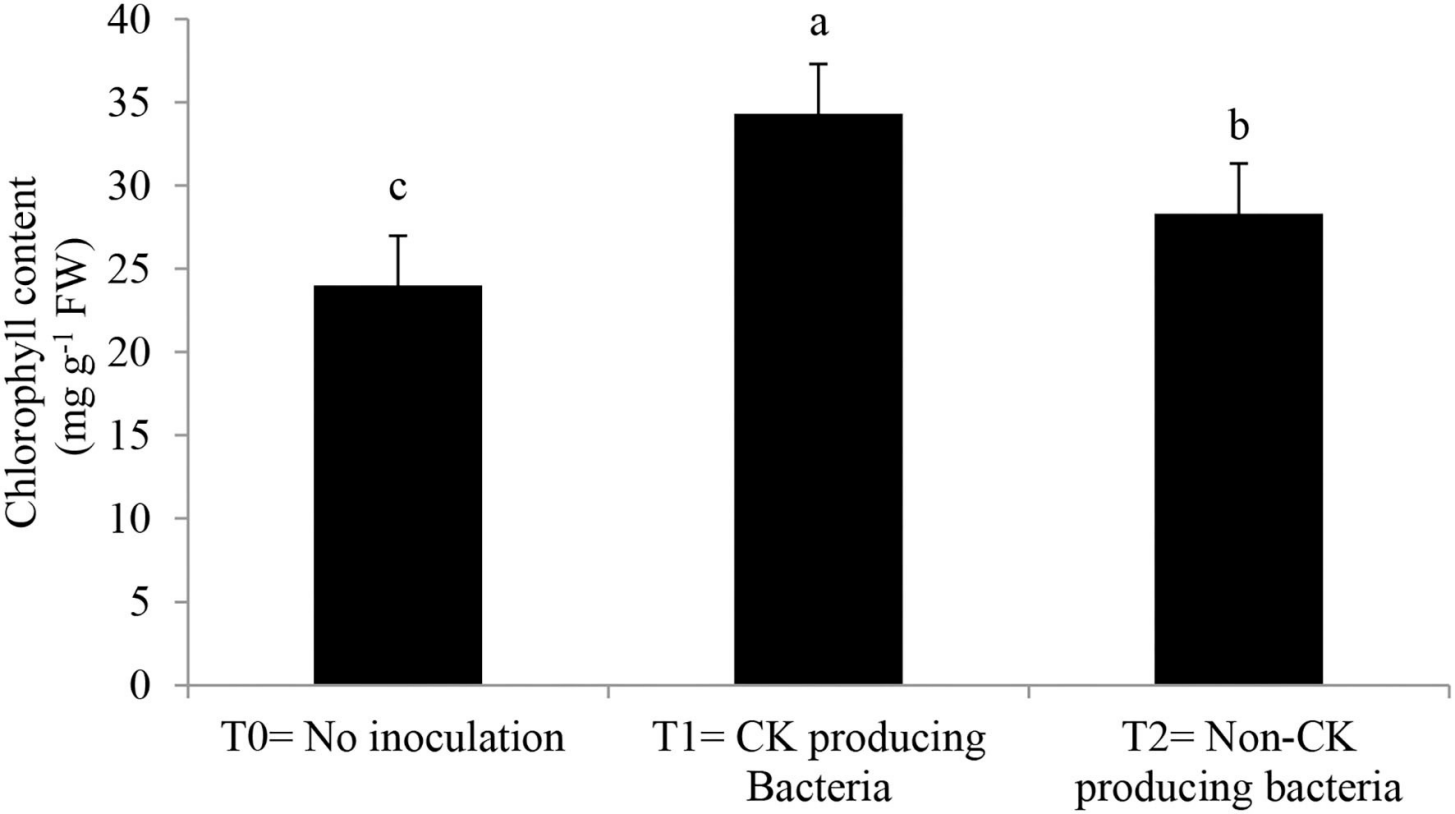
Effect of Ck producing *A. brasilense* RA−17 and non-producing *A. brasilense* RA−18 on relative chlorophyll content (mg g^−1^ FW) of wheat. T0 = no inoculation; T1 = Ck producing strain, and T2 = non-Ck producing bacterial strains. The different letter shows a significant difference in treatments at a 5% probability level.

**Figure 4:**
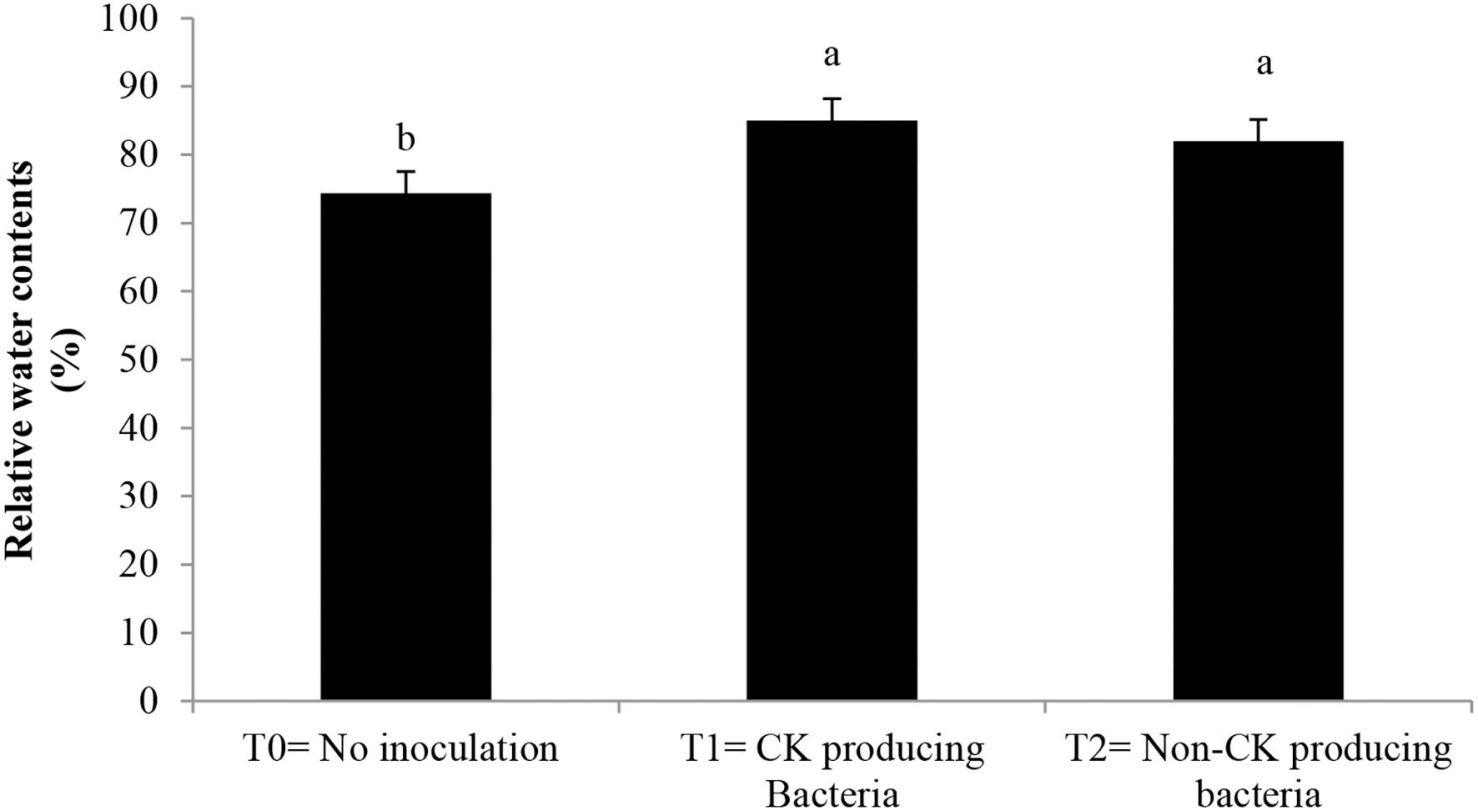
Effect of Ck producing *A. brasilense* RA−17 and non-producing *A. brasilense* RA−18 on relative water content (RWC, %) of wheat. T0 = no inoculation; T1 = Ck producing strain, and T2 = non-Ck producing bacterial strains. The different letter shows a significant difference in treatments at a 5% probability level.

Epicuticular wax, stomatal conductance, photosynthetic rate (Table 3), and chlorophyll contents (Figure 3) were greater with the Ck-producing than non-producing bacterial strain and were lowest in the uninoculated plants. RWC did not differ between plants inoculated with Ck-producing and non-producing bacterial strains but was lowest in the uninoculated plants (Figure 4).

**Table 3:** Effect of cytokinin-producing *A. brasilense* RA−17 (T_1_) and non-producing *A. brasilense* RA−18 (T_2_) on proline content, epicuticular wax, stomatal conductance, and photosynthetic rate of wheat at flag leaf stage.

**Treatments**	**Epicuticular wax** **(g cm^**−2**^)**	**Stomatal conductance** **(mmol m^**−2**^ s^**−1**^)**	**Photosynthetic rate** **(μ mol m^**−2**^ s^**−1**^)**
T_0_ (control)	0.007 c	266.33 c	7.84 c
T_1_	0.009 a	290.66 a	11.45 a
T_2_	0.008 b	279.00 b	9.84 b

*The different letter shows a significant difference in treatments at a 5% probability level*.

### Proline and Antioxidant Enzymes

**Figure 5:**
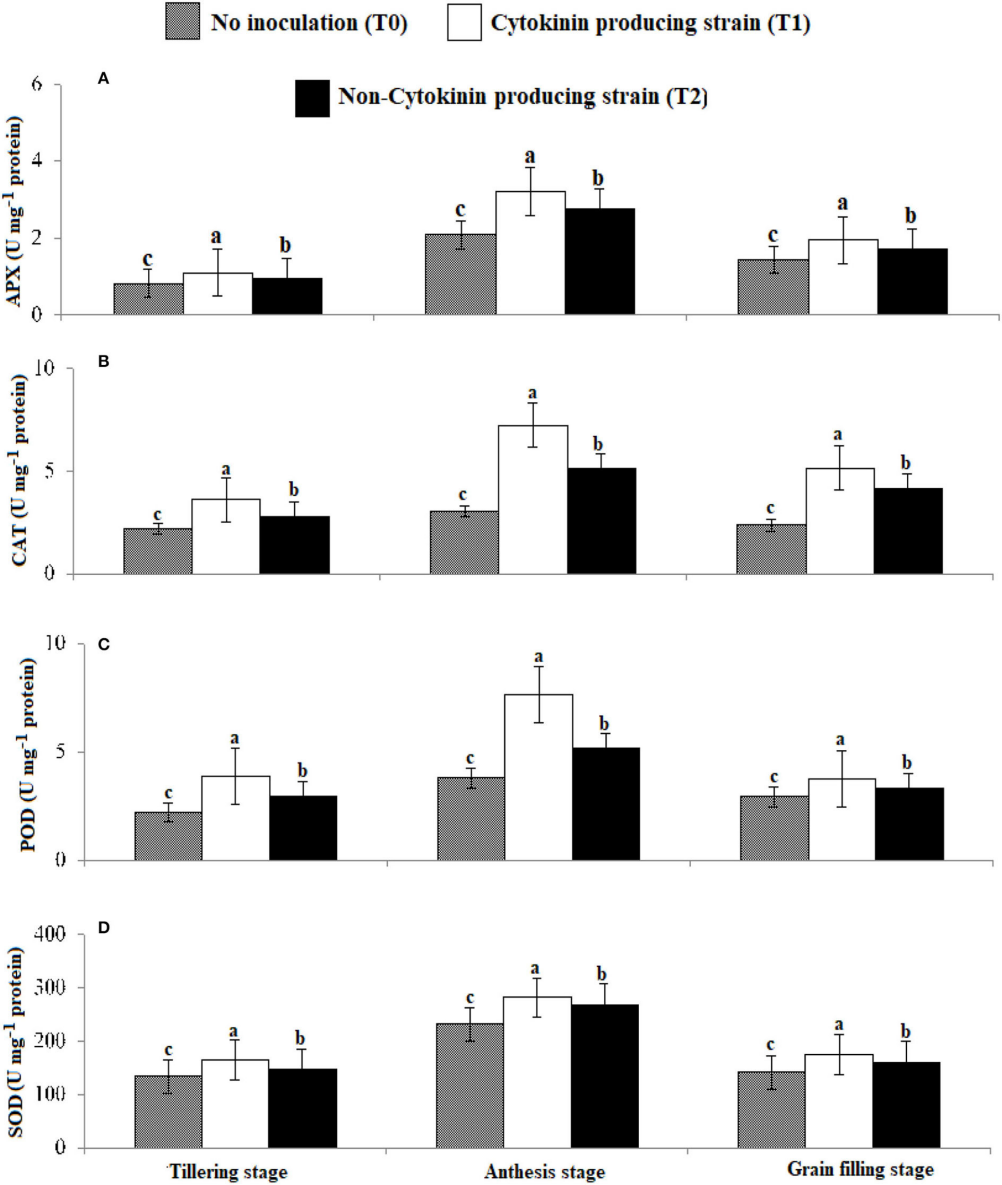
Effect of Ck-producing *A. brasilense* RA−17 and non-producing *A. brasilense* RA−18 on antioxidant enzymes activities (U mg^−1^ protein) of wheat. The different letter shows a significant difference in treatments at a 5% probability level.

**Figure 6:**
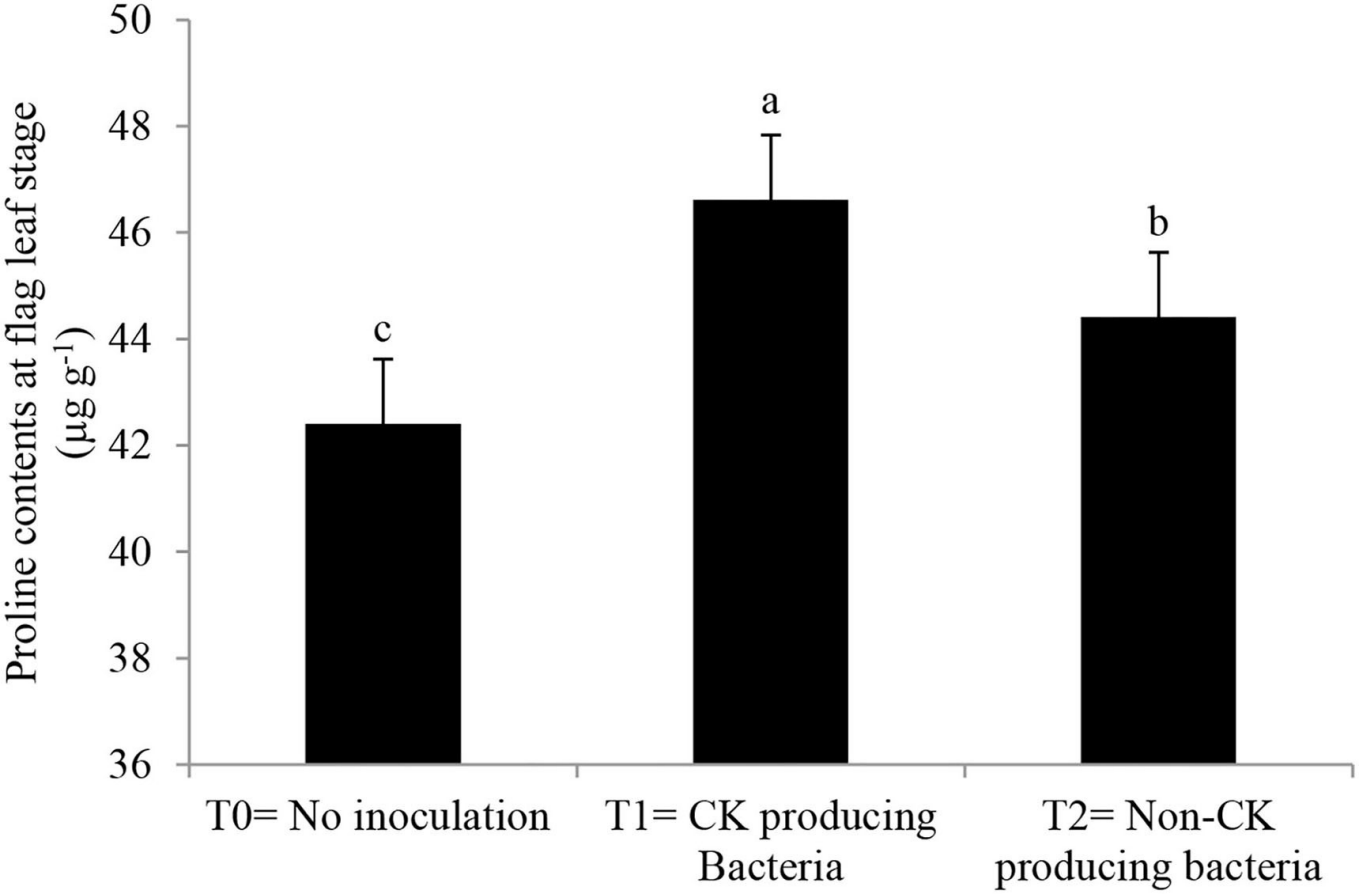
Effect of Ck-producing *A. brasilense* RA−17 and non-producing *A. brasilense* RA−18 on proline content at flag leaf stage of wheat. The different letter shows a significant difference in treatments at a 5% probability level.

Antioxidant enzyme activities and proline content (Figures 5, 6) were greater in those wheat plants that were inoculated with the Ck-producing compared with the non-producing strain and were lowest in the uninoculated plants. Generally, enzyme activities were highest at the anthesis stage and lowest at tillering stage.

### Endogenous Hormone Levels in Kernels

**Figure 7:**
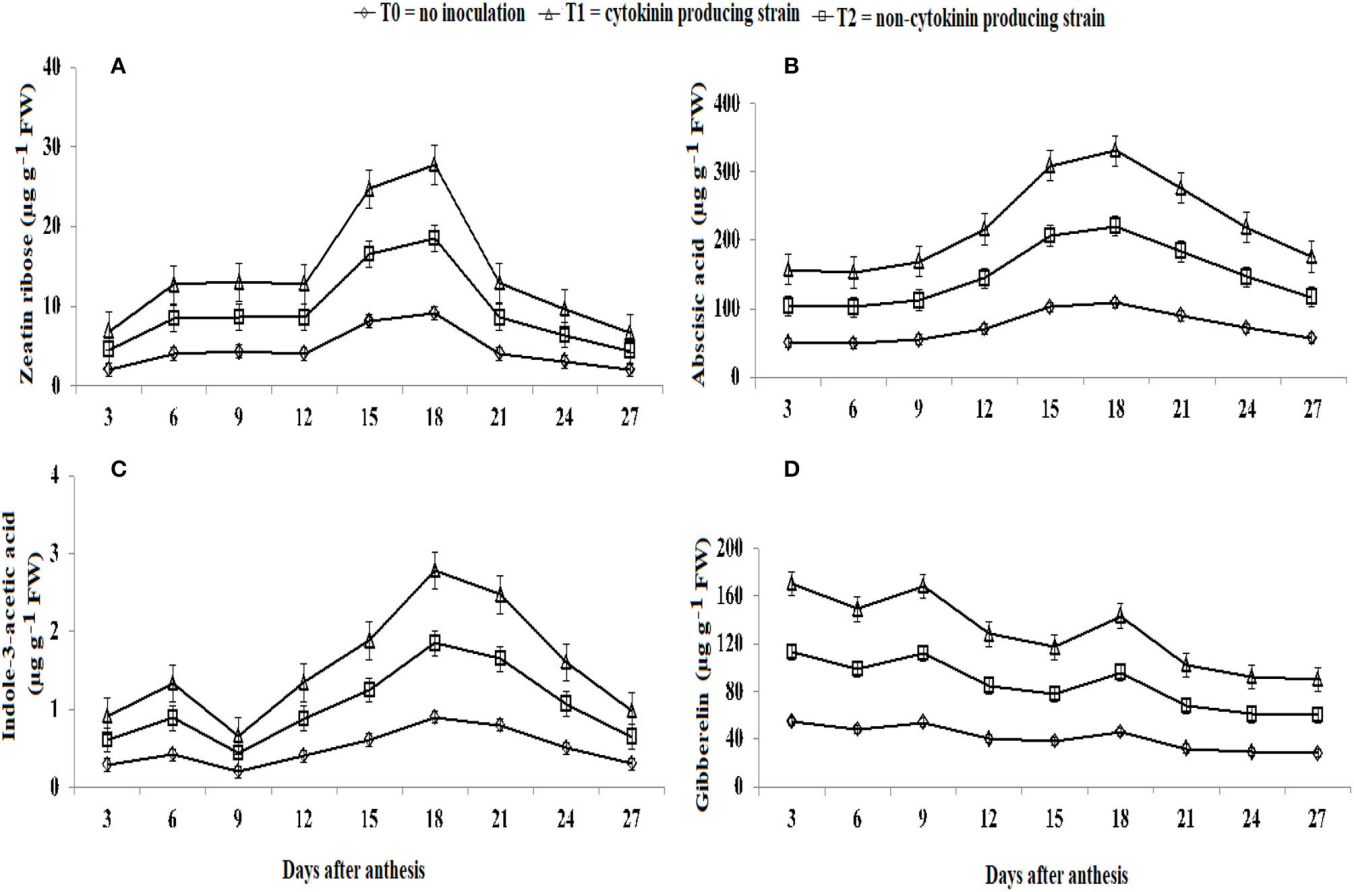
Effect of Ck-producing *A. brasilense* RA−17 and non-producing *A. brasilense* RA−18 on levels of endogenous hormones (μg g^−1^ FW) in wheat kernels.

The concentration of endogenous hormones in kernels of wheat was highest in wheat plants inoculated with the Ck-producing strain than non-producing strain and was lowest in the uninoculated plants (Figure 7). Zeatin riboside, IAA, and ABA levels increased from 3 to 18 days after anthesis and decreased sharply after (Figures 7A–C). Gibberellin level was high at the early grain filling stage and then decreased continually for 15 days and then increased from 15 to 18 days and then decreased again until 27.

## Discussion

### Influence of Ck-Producing Strain on Wheat Growth Parameters

*Azospirillum spp*. are known to be associated with several cereal crops (Ibrahim et al., 2016; Chávez-Herrera et al., 2018; Zaheer et al., 2021a). In this study, inoculation of wheat seed with strains of *A. brasilense* increased plants' growth parameters (plant height, CGR, RGR, NAR, and LAI) at different developmental stages than in uninoculated control. Seed inoculation with rhizobacteria has improved plant growth performance by releasing phytohormones, such as IAA, Ck, and gibberellins (Lakshmanan et al., 2013). For example, in grapevines, *A. brasilense* Sp245 enhanced the root and vegetative development (Bartolini et al., 2017), cucumber seedlings (Pereyra et al., 2010), and wheat plants (Spaepen et al., 2008) by producing IAA, and *A. brasilense* improved growth and photosynthetic parameters in wheat by producing Cks (Afzal et al., 2014; Zaheer et al., 2019b).

However, in this study, wheat inoculation with strain RA−17 produced a higher level of Ck significantly increased growth parameters compared with strain RA−18. Cks are phytohormones that influence plant growth and development by cell division, expansion of leaves, delay leaf senescence, etioplasts conversion to chloroplasts, and chlorophyll accumulation in leaves (Zafar et al., 2012; Zwack and Rashotte, 2013). Ck-producing *Magnaporthe oryzae* aided plant nutrient mobilization, increased photosynthetic levels and activated salicylic acid-mediated defense response in rice (Chanclud et al., 2016). Whereas, the Ck-mutant with a deleted Ck synthesizing gene did not show distinct growth and development features until an exogenous source of Ck was applied. In a study by Esquivel-Cote et al. (2010), inoculation of tomato seeds with a higher Ck-producing *Azospirillum* strain, *A. lipoferum* AZm5, enhanced RGR and increased leaf area, thus resulting in an increased photosynthetic surface, compared with a low-Ck producing strain VS9. However, they found that NAR did not increase due to nitrogen deprivation, which was reversed after the supply of N-fertilizer.

### Influence of Ck-Producing Strain on Wheat Yield Parameters

All yield-promoting parameters are more significant with the Ck-producing than non-producing strain and were lowest in the uninoculated plants. Yield improvement in rhizobacteria-inoculated plants is due to improved water uptake and nutrient availability (Liu et al., 2019). In Panda et al. (2018), Ck significantly improved grain filling by increasing the expression of cell cycle regulators in the spike's basal spikelets, leading to enhancement in grain filling. Mohapatra et al. (2011) showed the growth promotion effect of Ck in rice with the improving grain filling in rice panicles. Ck production by rhizobia was also shown to enhance the growth of different crops (Hayat et al., 2008). Barley inoculation with Ck-producing *A. brasilense* Sp246 increased yield and related parameters compared with the uninoculated control (Ozturk et al., 2003). Saber et al. (2012) also reported higher grains in wheat inoculated with plant growth-promoting rhizobacteria that lead to a higher yield.

### Influence of Ck-Producing Strain on Proline Content and Antioxidant Enzyme Activities

Inoculating wheat seeds with the Azospirillum strains increased proline content and antioxidant enzyme activity (APX, CAT, POD, and SOD) more than in uninoculated plants. However, inoculation with high Ck-producing *Azospirillum* strain AR−17 than strain AR−18 increased the accumulation of these metabolites, particularly at anthesis. Rhizobacteria produce different metabolites to enhance stress tolerance and remove the adverse effects of reactive oxygen species (ROS) in plants (Madhaiyan et al., 2006; Raheem et al., 2018; Zaheer et al., 2021a). Synthesis of proline is increased by upregulation of the P5CS gene in the presence of Ck and ABA in plants (Vacheron et al., 2013). Different Ck-producing rhizobacterial strains have been noted to increase proline content under salt stress in soybean (Naz et al., 2009). Higher activities of APX, CAT, POD, and SOD were noticed at tillering, anthesis, and grain filling stages with the strain AR−17 than strain AR−18. As opined by Naseem et al. (2014), Ck can modulate defense signaling by activating antioxidant enzymes. Argueso et al. (2012) demonstrated that higher levels of Ck increased immune defenses in Arabidopsis. They showed that the enhanced levels of Ck gave higher protection in wild Arabidopsis plants. Ghorbanpour et al. (2013) and El-Esawi et al. (2018) reported that rhizobacterial inoculation of *Hyoscyamus niger* improves antioxidant enzymes activities, thus activating the plant defense mechanism. Chang et al. (2016) observed that levels of antioxidant enzymes increased in plants when Ck level was also increased.

### Influence of Ck-Producing Strain on Endogenous Hormone Levels in Wheat Kernels

Inoculation with Ck-producing *Azospirillum* strain AR−17 increased the contents of zeatin riboside, gibberellin, IAA, and ABA, compared with strain AR−18, in wheat kernels and was lowest in the uninoculated plants. Higher hormonal contents in the kernels have been noted to significantly increase grain filling percentage (Yang et al., 2000; Zhang et al., 2010) and also regulate the sink size of the kernels (Yang et al., 2003). However, a study of Ck application in wheat kernels is limited. Yang et al. (2016) found that the contents of zeatin riboside, gibberellin, IAA, and ABA were significantly correlated with grain-filling rate. Production of endogenous hormones is essential for the regulation of kernel development. For example, zeatin maximizes the endosperm cell division, and increases sink capacity, thus increasing assimilating accumulation (Yang et al., 2002). An increase in IAA content could reverse the reduction in grain weight (Yan et al., 2005). This suggests that Ck could sustain a more active photosynthetic period, thus transferring more assimilates to the grains during the grain filling stage, (Chen et al., 2010), and finally increase yield.

## Conclusion

*Azospirillum brasilense* inoculation with the wheat seed significantly affected the wheat crop's growth, yield, and physiological parameters. Ck-producing *A. brasilense* is more beneficial for the growth, yield, antioxidant, and physiological systems of wheat than the non-Ck-producing *A. brasilense*. Ck producing *A. brasilense* is very effective for higher antioxidant enzyme activities and growth hormones production in wheat crops.

## Data Availability Statement

The original contributions presented in the study are included in the article/supplementary material, further inquiries can be directed to the corresponding author/s.

## Author Contributions

MZ, MM, and KE planned and executed the field experiment. HA and MI supervised the investigation. MZ, MM, KE, and MI participated in a write-up and review of the manuscript. MZ, TJ, and JI performed the antioxidant analysis. MH, ES, HK, JW, and ED reviewed the article and performed a revision of the manuscript. All authors made critically reviewed the manuscript, approved the final version, substantial contributions to the acquisition, analysis, and interpretation of the data described in this study.

## Funding

The current work was funded by Taif University Researchers Supporting Project number (TURSP−2020/85), Taif University, Taif, Saudi Arabia.

## Conflict of Interest

The authors declare that the research was conducted in the absence of any commercial or financial relationships that could be construed as a potential conflict of interest.

## Publisher's Note

All claims expressed in this article are solely those of the authors and do not necessarily represent those of their affiliated organizations, or those of the publisher, the editors and the reviewers. Any product that may be evaluated in this article, or claim that may be made by its manufacturer, is not guaranteed or endorsed by the publisher.
